# Co-culture of induced pluripotent stem cells with cardiomyocytes is sufficient to promote their differentiation into cardiomyocytes

**DOI:** 10.1371/journal.pone.0230966

**Published:** 2020-04-03

**Authors:** Axel J. Chu, Eric Jiahua Zhao, Mu Chiao, Chinten James Lim

**Affiliations:** 1 School of Biomedical Engineering, The University of British Columbia, Vancouver, B.C., Canada; 2 Michael Cuccione Childhood Cancer Research Program, BC Children’s Hospital Research Institute, Vancouver, B.C., Canada; 3 Department of Mechanical Engineering, The University of British Columbia, Vancouver, B.C., Canada; 4 Department of Pediatrics, The University of British Columbia, Vancouver, B.C., Canada; University of Tennessee Health Science Center College of Medicine Memphis, UNITED STATES

## Abstract

Various types of stem cells and non-stem cells have been shown to differentiate or transdifferentiate into cardiomyocytes by way of co-culture with appropriate inducer cells. However, there is a limited demonstration of a co-culture induction system utilizing stem cell-derived cardiomyocytes as a stimulatory source for cardiac reprogramming (of stem cells or otherwise). In this study, we utilized an inductive co-culture method to show that previously differentiated induced pluripotent stem (iPS) cell-derived cardiomyocytes (iCMs), when co-cultivated with iPS cells, constituted a sufficient stimulatory system to induce cardiac differentiation. To enable tracking of both cell populations, we utilized GFP-labeled iPS cells and non-labeled iCMs pre-differentiated using inhibitors of GSK and Wnt signaling. Successful differentiation was assessed by the exhibition of spontaneous self-contractions, structural organization of α-actinin labeled sarcomeres, and expression of cardiac specific markers cTnT and α-actinin. We found that iCM-iPS cell-cell contact was essential for inductive differentiation, and this required overlaying already adherent iPS cells with iCMs. Importantly, this process was achieved without the exogenous addition of pathway inhibitors and morphogens, suggesting that ‘older’ iCMs serve as an adequate stimulatory source capable of recapitulating the necessary culture environment for cardiac differentiation.

## Introduction

One of the most adopted methods for generating cardiomyocytes (CMs) from pluripotent stem cells is by pharmacological manipulation [1–3]. Another strategy is by culturing stem cells with the appropriate cell or tissue-based inducer/s [4, 5]. The latter approach stems from the *a priori* assumption that one can overcome the complexity of precisely recapitulating the biochemical signaling events associated with cardiac organogenesis by relying on already differentiated CMs or other cells found in the cardiac microenvironment. However, this approach is not without theoretical flaws. CMs that have been terminally differentiated or are not stimulated by ischemia / injury may not produce the necessary signaling cues essential to cardiac differentiation [6]. Moreover, the perceived plasticity of cultured stem cells in *in vivo* transplantation may be attributed to an entirely different set of milieu-dependent differentiation mechanisms that may be impossible to recreate in an *in vitro* setting [7]. Despite these limitations, there have been documented successes in efforts to derive CMs from other cell types (stem cells or otherwise) by inductive co-cultures.

One of the first reported successes of creating CMs from human pluripotent stem cells via co-culture induction came from Mummery *et al*. [8]. They showed that co-culture of human embryonic stem (ES) cells with mouse visceral-endoderm-like cells initiated differentiation into spontaneously contracting cells. Although the exact mechanism is still unknown, bone morphogenetic proteins (BMPs), fibroblasts growth factors (FGFs), and inhibitors of Wnt produced by endodermal cells are likely players involved in the process. Similarly, when Rudy-Reil *et al*. co-cultured murine ES cells with a bilayer of avian precardiac endoderm / mesoderm, the number of contractile embryoid bodies was significantly increased compared to cells cultured alone [9]. More recently, Ou *et al*. demonstrated that the long-term differentiation of mouse ES cells into CMs proved more efficient when co-cultured with mouse neonatal CMs compared to sole treatment with ascorbic acid to induce cardiac differentiation [10]. In a subsequent study, the authors concluded that co-culture of stem cells with neonatal CMs induced genes to be expressed in a mature pattern and stimulated the proliferation of stem cell-derived CMs by activating FAK/JNK signaling [11].

Examples of transdifferentiation of already differentiated cells into CMs have also been documented. Most notable was the report by Condoelli *et al*. showing rat endothelial cells, freshly isolated from embryonic vessels, changing fate and transdifferentiating into beating CMs after being co-cultured with neonatal rat CMs [12]. Badorff *et al*. corroborated this finding by observing the transdifferentiation of endothelial progenitor cells, obtained from peripheral blood mononuclear cells of healthy adults and coronary heart disease patients, into CMs after co-cultivation with rat CMs [13].

In the present work, we evaluated if an inductive co-culture system utilizing induced pluripotent stem (iPS) cell-derived cardiomyocytes (iCMs) is a sufficient stimulatory source for cardiac reprogramming of iPS cells. Such a system may enable personalized cardiac tissue regeneration that can supplant the use of exogenous pharmacological agents as programming factors with unknown long-term effects on CMs.

## Materials and methods

### Cell lines

The iPS cell line, IMR90 (WiCell Research Institute, Inc), was generated from lung fibroblasts obtained from a 16-week old human female fetus using viral transduction of the *OCT4*, *NANOG*, *SOX2*, and *Lin-28 homolog A (LIN28)* genes. Upon receipt of the IMR90 iPS cells, they were exclusively cultured in mTeSR1 medium (Stem Cell Technologies) and on Matrigel (Corning) coated surfaces.

AICS16 and AICS11 are human clonal iPS cell lines made by the Allen Institute for Cell Science (Coriell Institute) in which a single allele of *ACTB (beta actin)* or *TOMM20*, respectively, was tagged as a *monomeric enhanced green fluorescent protein (mEGFP)*-fusion protein. The GFP_+ve_ AICS16 and AICS11 cells were used to track cardiac differentiation outcomes of iPS cells co-cultured with non-labeled iPS (IMR90) cell-derived cardiomyocytes (iCMs).

### Cardiac differentiation with GSK3 inhibitor and Wnt inhibitor (GiWi protocol)

**Fig 1:**
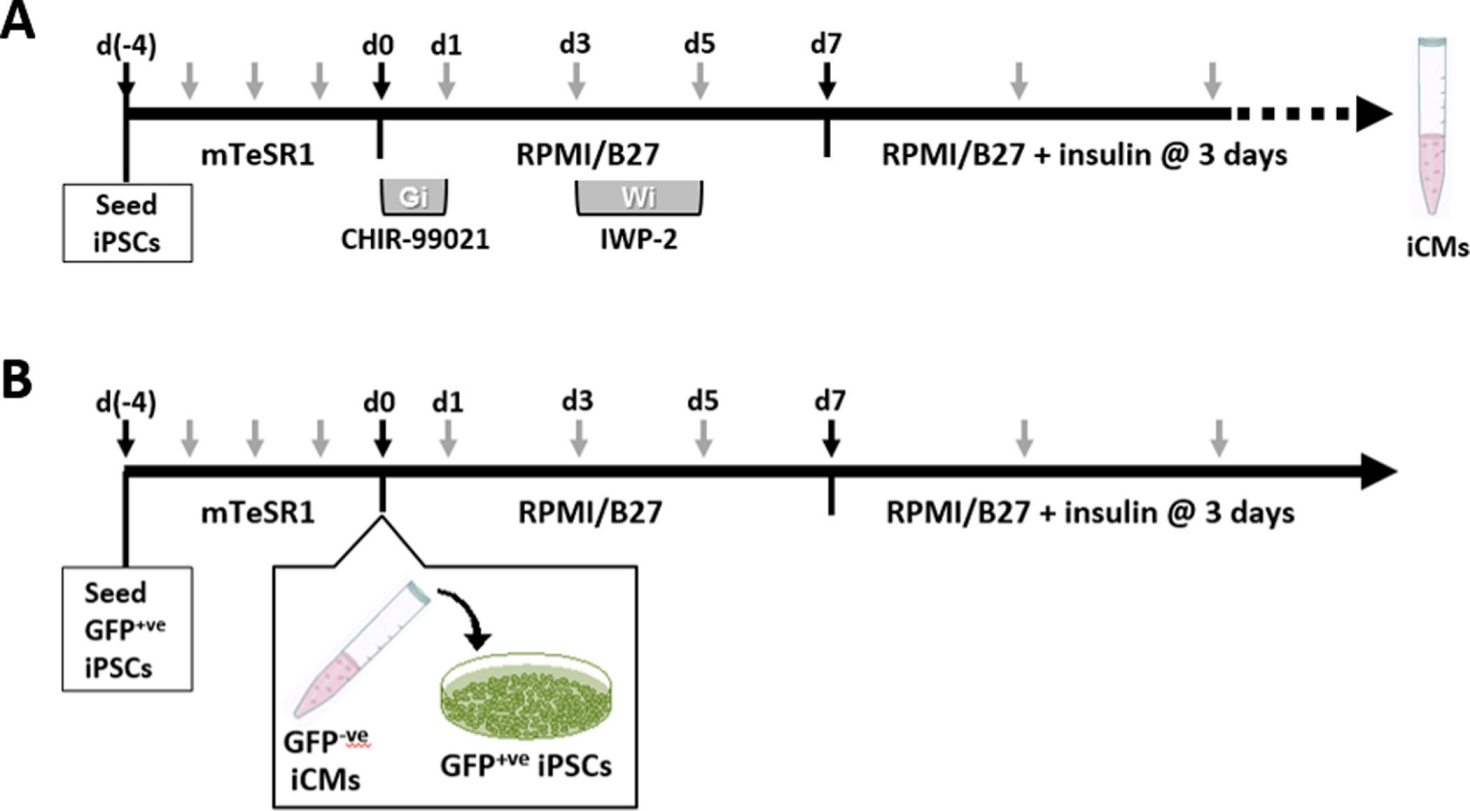
Schematic of GiWi and co-culture method used to initiate cardiac differentiation of iPS cells. (A) For differentiation by GiWi, iPS cells were seeded onto Matrigel-coated plates and cultured to ~90% confluence in mTeSR1. The basal media was switched to RPMI/B27 at days 0–7, with addition of CHIR-99021 at days 0–1, and IWP-2 at days 3–5. Thereafter, the basal media was switched RPMI/B27/insulin. (B) For co-culture differentiation, GFP_+ve_ iPS cells (AICS11 or AICS16) were similarly cultured to ~90% confluence in mTeSR1. At day 0, iCMs derived from non-labeled GiWi-differentiated iPS cells were dissociated and added to GFP_+ve_ iPS cells. Cells are maintained in RPMI/B27 at days 0–7 (without GiWi), and subsequently in RPMI/B27/insulin. Black arrows indicate key steps, while gray arrows indicate the frequency of culture media replacement.

To generate cardiomyocytes from iPS cells via traditional biochemical means, we employed the GiWi protocol [1], involving inhibition of glycogen synthase kinase 3 (GSK3) and Wnt (schematic shown in Fig 1A). iPS cells were seeded on Matrigel-coated 6-well plates at a density of 1×10^5^ cells per well and maintained in mTeSR1 medium with daily medium renewal. When cells reached ~90% confluency, the media was changed to RPMI/B27 (RPMI 1640 basal medium (Sigma-Aldrich #R8758) with B-27 supplement minus insulin (Life Technologies #A1895601)). The GSK-3α/β inhibitor, CHIR-99021 (Selleck Chemicals #S2924), was added at 12μM for 24 hours, following which the medium was replaced with RPMI/B27 for 48 hours. Then, the medium was replaced with RPMI/B27 supplemented with the Wnt inhibitor IWP-2 (Stemcell Technologies #72122) at 5μM for 48 hours. Finally, the medium was replaced with RPMI/B27/insulin (RPMI 1640 with B-27 supplement complete with insulin (Life Technologies #17504–044)) and refreshed every 3 days thereafter. Spontaneous contractions are typically observed 7 days after the onset of CHIR-99021 treatment. With continued maintenance, the iPS cell-derived cardiomyocytes, now referred to as iCMs, remodel and recruit more cells to form larger contracting networks.

### Inductive co-culture differentiation of CellTracker^TM^-labelled iPS cells using pre-culture or bi-culture methods

Two days prior to the co-culture, IMR90 iPS cells in Matrigel-coated 6-wells were labeled with the CellTracker^TM^ Red CMTPX fluorescent dye (ThermoFisher Scientific #C34552). Separately, unlabeled IMR90 iPS cells were differentiated into iCMs. iCMs were harvested using 0.05% trypsin, neutralized with 10% FBS/RPMI (RPMI 1640 basal medium with 10% fetal bovine serum), pelleted, resuspended in RPMI/B27 and counted. In the pre-culture method, 3×10^6^ iCMs were added to each well of the CellTracker-labelled and adherent iPS cells. In the bi-culture method, CellTracker-labelled iPS cells were also harvested in similar fashion to the iCMs, mixed at equal numbers of iPS:iCM cells, then seeded onto Matrigel-coated plates. Day 0 was defined as the day iCMs and iPS cells were combined in the co-culture. The co-cultures were then maintained in RPMI/B27 for 7 days with medium renewed on days 1, 3, and 5. On day 7, the medium was changed to RPMI/B27/insulin, with imaging of the co-cultures performed at day 8 and thereafter using a fluorescent microscope. On day 8, pre-cultures were also harvested and reseeded at low density (5×10^5^ cells) onto 25mm Matrigel-coated glass cover slips and allowed to adhere for 24 hours before imaging.

### Inductive co-culture differentiation of GFP-tagged iPS cells using pre-culture method

This procedure is identical to that described above with the exception that the iPS cells, AICS11 or AICS16, stably express GFP-fusion proteins and do not require additional labeling. The iCMs are IMR90 iPS cells that was GiWi-differentiated for 30–45 days, trypsin harvested, and 3×10^6^ iCMs overlayed onto AICS11 or AICS16 iPS cell cultures at 90% confluency on Matrigel in 6-wells. The co-culture was then maintained in RPMI/B-27 for 7 days with medium renewal on days 1, 3, and 5. On day 7, the medium was switched to RPMI/B-27/insulin and medium renewed every 3 days. Co-cultures were maintained for 23–25 days prior to harvesting for downstream assays. Fig 1B outlines a schematic of the co-culture incubation scheme used to initiate cardiac differentiation of the GFP_+ve_ AICS11 and AICS16 iPS cells. For each experiment, a non-co-culture control was setup in parallel, where AICS11 or AICS16 cells received the same media changes but without the overlay of iCMs.

### Microscope systems

Cell images were acquired on an Olympus IX81 inverted fluorescent microscope equipped with differential interference contrast (DIC) objectives, a CoolSNAP HQ2 camera (Photometrics), and an environmental chamber (temperature, humidity and CO_2_) that allowed both live and fixed cell experiments. The X-Cite exacte illuminator (Lumen Dynamics) was used as a light source with excitation and emission bandpass filters for visualizing blue, green, and red fluorescent markers. MetaMorph software (v. 7.8) was used for data acquisition and post-acquisition analysis was performed using ImageJ (NIH). Additional imaging was obtained with the Olympus CKX31 inverted microscope equipped with an eyepiece mounted digital camera (Novel Optics #HDCE-20C).

### Kymograph generation and analysis

Kymographs are graphical representation of spatial position over time in which the spatial axis represents time. It allows rhythmic motion, such as spontaneous self-contractions of cardiomyocytes, to be computed by performing a selected line scan for every frame in a video clip. From the periodic motions of spontaneously beating cardiomyocytes, peaks of contraction could be rendered. The beat frequency (beats per unit time) of contracting cardiomyocytes and their clusters were calculated by dividing the total number of peaks by total time. Kymographs were generated using a built-in tool within ImageJ.

### Immunocytochemistry

For the purpose of visualizing sarcomeric α-actinin, multicellular tissue aggregates were harvested and reseeded at low single cell density onto glass coverslips. For dissociation, cells were rinsed with phosphate-buffered saline (PBS) (Sigma-Aldrich #D8537) before treatment with 1mL trypsin-ethylenediaminetetraacetic acid (EDTA) solution (0.05% trypsin, GE Healthcare #SH30236.01) at 37°C for ~5 minutes to dissociate iPS cells, and up to ~9 minutes to dissociate iCMs. Next, trypsin was neutralized with 2mL 10% FBS/RPMI, and cells further disaggregated by repeated pipetting. Cells were pelleted by centrifugation at 300g for 5 minutes and resuspended in appropriate medium (mTeSR1 for iPS cells and RPMI/B27/insulin for iCMs).

Typically, ~5×10^5^ cells were seeded onto Matrigel-coated 25mm circular coverslips (Fisher Scientific #12-545-102) in 6-well plates and incubated at 37°C for 24 hours to promote adhesion. Subsequently, cells were washed with PBS and fixed in 3.7% formaldehyde/PBS for 15 minutes (Fisher Scientific #BP531-500)) and permeabilized with 0.1% triton X-100/PBS (Sigma-Aldrich #9002-93-1) for 5 minutes, at 22°C. Cells were blocked with 1%BSA/PBS for 20 minutes, and incubated with anti-α-actinin antibody (Sigma-Aldrich #A7811) at 1:800 dilution in PBS overnight at 4°C. Following washes with PBS, cells were incubated with DyLight633-conjugated goat anti-mouse IgG (ThermoFisher Scientific #35513) at 1:250 dilution for 30 minutes. Following washes with PBS, cells were mounted onto glass slides using ProLong Gold with DAPI (Life Technologies #P-36931) for imaging.

### Flow cytometry

To prepare cells for flow-based analysis, cells harvested using trypsin was fixed and permeabilized by resuspension in 250μL BD Cytofix/Cytoperm™ solution (BD Biosciences #554722) for 20 minutes at 4°C. Cells were washed twice with 1mL BD Perm/Wash™ buffer (BD Biosciences #554723) and incubated with anti-cardiac troponin T (anti-cTnT) (ThermoFisher Scientific #MA5-12960) and anti-α-actinin (Sigma-Aldrich #A7811) at dilutions of 1:100, 1:800 respectively for 30 minutes at 4°C. Following 2 washes with BD Perm/Wash buffer, cells were labeled with DyLight633-conjugated goat anti-mouse IgG (ThermoFisher Scientific #35513) at 1:250 dilution for 30 minutes. Following 2 more washes, cells were resuspended in 200μL of BD Perm/Wash buffer, and flow data acquired using the BD Accuri™ C6 flow cytometer set for 30,000 events. Post-acquisition analysis was conducted using FlowJo v10.2 (BD Biosciences).

To calculate differentiation efficiency, the following scheme was applied. Flow data was gated into 4 quadrants, with Q1 and Q4 designated as GFP_-ve_ cells, while Q2 and Q3 are GFP_+ve_ cells. In addition, cells in Q1 and Q2 are designated as cardiomyocyte populations. Thus, the % differentiation efficiency of GFP_+ve_ cells is calculated as Q2/(Q2+Q3)*100; while general differentiation efficiency (label independent) is calculated as (Q1+Q2)/(Q1+ Q2+Q3+Q4)*100.

### Statistical analysis

Statistical significance was determined using ordinary one-way ANOVA followed by Tukey’s multiple comparisons test using GraphPad Prism 8.

## Results

### iPS cells co-cultured with iCMs exhibit spontaneous self-contractions

**Fig 2:**
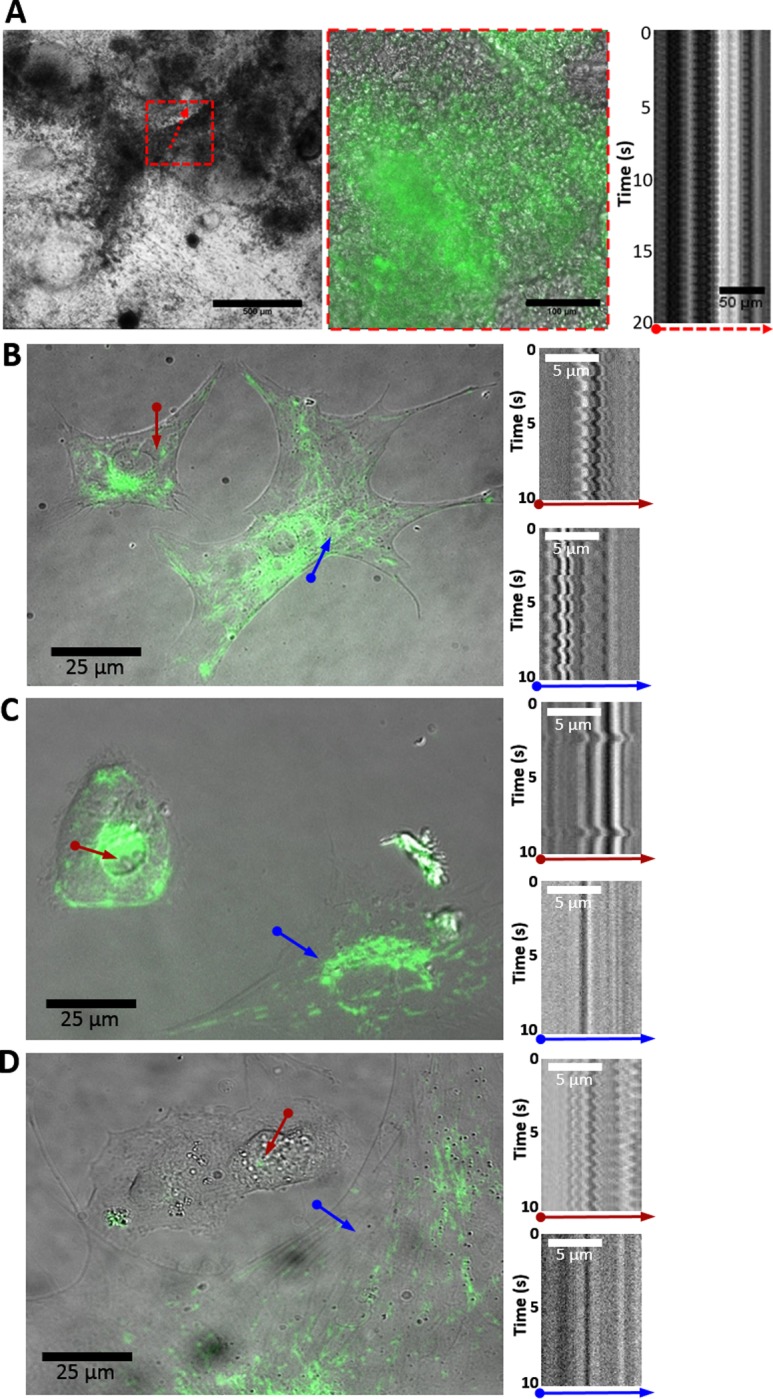
AICS11 and AICS16 iPS cells co-cultured IMR90 iCMs exhibit spontaneous self-contractions. (A) DIC image of pre-culture on day 23 (scale bar: 500μm), red box shows magnified overlay of DIC and GFP images (scale bar: 100μm). Kymograph of contracting cluster indicated by red arrow (scale bar: 50μm). (B-D) Co-cultures were dissociated and reseeded at low cell density for imaging and kymograph analysis of isolated cells. Kymogram traces are for the indicated colored arrows. In (D), a non-contractile GFP_+ve_ cell (blue arrow) is shown in proximity to a GFP_-ve_ cell exhibiting self-contractions (red arrow). Scale bars: 25μm in overlays; 5μm in kymographs. Cells are AICS16 for A; AICS11 for B,C,D.

To evaluate our hypothesis that iPS cells can be differentiated into cardiomyocytes when co-cultured with iCMs, we co-cultured IMR90 iPS cells with IMR90-derived iCMs. The IMR90 iCMs were generated using the so-called ‘GSK inhibitor-Wnt inhibitor (GiWi) protocol’ [1], in which small molecule inhibitors of glycogen synthase kinase 3 (GSK3) and Wnt were applied to an iPS cell monolayer on days 0 and 3, respectively (Fig 1A). To enable tracking of cells differentiated as a consequence of the co-culture, iPS cells were labeled with CellTracker™ Red, a fluorescent dye that facilitates tracking of the labeled cell for up to eleven days. Two seeding methods were considered: 1) Pre-culture method, in which cells were seeded sequentially (Fig 1A), and 2) Bi-culture method, in which cells were seeded simultaneously. Video analysis on day 9 of both co-culture methods revealed contractile masses containing both labeled and non-labeled cells, but could not achieve single-cell resolution for contractility (S1 and S2 Figs). To assess this, the co-cultures were dissociated and replated at low density onto coverslips for single cell imaging and kymograph analysis. We found that the pre-culture method yielded fluorescently-labeled cells that exhibit self-contractions (S1 Fig), while the bi-culture method yielded no such cells (S2 Fig). This initial success suggested the pre-culture method facilitated the successful differentiation of iPS cells into CMs, enabled by an overlay co-culture with existing iCMs. However, CellTracker suffered from diminishing fluorescence after ~10 days post labelling, which prevented longer term evaluation of programming efficiency of the iPS cells.

Therein, we extended application of the pre-culture method to include the human iPS cell lines, AICS11 and AICS16, which stably express TOMM20 and beta-actin, respectively, as GFP-fusion proteins. This allowed for the visualization and tracking of the cells spatially and temporally, without the limitation of fluorescent loss due to metabolic breakdown or cell division-mediated dilution of the dye-label. Using the pre-culture method, iCMs (GiWi-differentiated from non-labeled IMR90) were seeded onto GFP_+ve_ AICS11 or AICS16 iPS cells (Fig 1B). Successful reprogramming of iPS cells to cardiomyocytes was assessed on the basis of GFP_+ve_ cells exhibiting spontaneous self-contractions, which is one of the key functional attributes of stem cell-derived cardiomyocytes [14].

Image analysis of co-cultures carried out on day 23 revealed extensive networks of spontaneously contracting clusters with GFP_+ve_ cells integrated into and concentrated at the contracting nodes (Fig 2A). A kymograph analysis of the contracting clusters revealed periodic perturbations indicative of rhythmic motion, characteristic of spontaneous cellular contractions. To detect spontaneous self-contractions of GFP_+ve_ cells in isolation, the co-cultures were harvested on day 25 and reseeded at low density onto Matrigel-coated glass slips, and imaged the following day at higher magnification. Singular GFP_+ve_ cells can be seen to exhibit spontaneous self-contractions (Fig 2B and 2C) interspersed with other GFP_+ve_ cells that were not contracting (Fig 2C and 2D), and as well with contractile GFP_-ve_ cells (Fig 2D). The observation of GFP_+ve_ cells sustaining spontaneous self-contraction provides supporting evidence that the incubation of iPS cells in a co-culture manner with previously differentiated iCMs constitutes a sufficient system to induce cardiac differentiation of iPS cells.

### Co-cultured GFP-tagged cells exhibit α-actinin sarcomeric striations

Another defining characteristic of cardiomyocytes is the expression of structural proteins, such as α-actinin, and the organization of these proteins into sarcomeres [14]. To characterize the structural maturity of resulting iCMs generated via co-culture incubation method, immunocytochemistry staining for α-actinin protein was performed and maturity was assessed by the presence of sarcomeric striations. The co-cultured cells were also compared to GFP_+ve_ iPS cells that were differentiated using the GiWi protocol (positive control) and GFP_+ve_ iPS cells that were subjected to only basal medium + supplement changes with no differentiation factors added (negative control).

**Fig 3:**
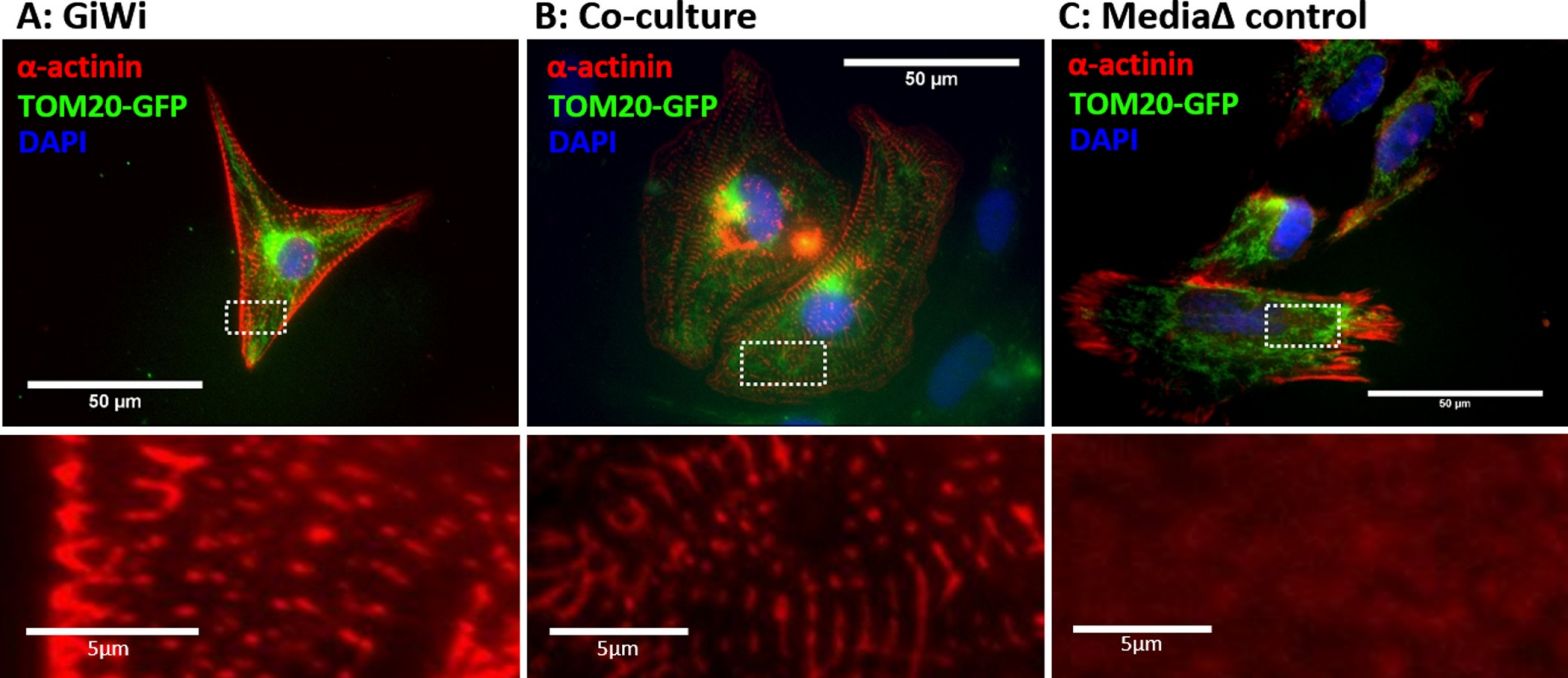
Staining of AICS11 (TOM20-GFP) cells for sarcomeric α-actinin. AICS11 cells were differentiated using (A) GiWi protocol or (B) inductive co-culture with iCMs, and (C) basal media change alone (absent differentiation factors). Bottom panels show a magnified image of α-actinin staining for the area bounded by white rectangles. Scale bars are 50um for top panels and 5um for bottom panels.

**Fig 4:**
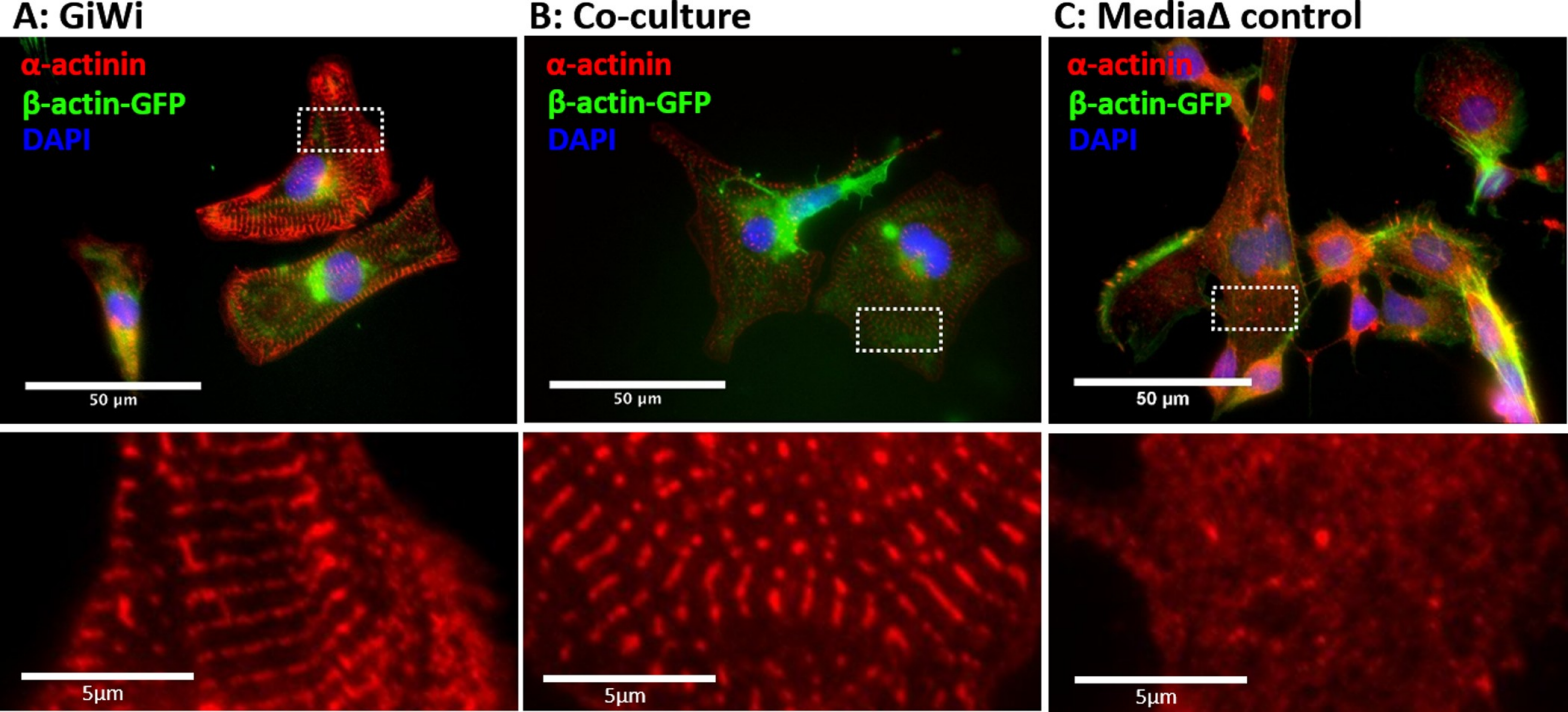
Staining of AICS16 (β-actin-GFP) cells for sarcomeric α-actinin. AICS16 cells were differentiated using (A) GiWi protocol or (B) inductive co-culture with iCMs, and (C) basal media change alone (absent differentiation factors). Bottom panels show a magnified image of α-actinin staining for the area bounded by white rectangles. Scale bars are 50um for top panels and 5um for bottom panels.

Analysis of images revealed the distinct formation of sarcomeric striations in AICS11 GFP_+ve_ cells that were treated with the GiWi protocol (Fig 3A) and those that were incubated using the co-culture method (Fig 3B), but not in cells subjected to media change alone (Fig 3C). The presence of sarcomeric striations in co-cultured GFP_+ve_ cells is indicative of cells having mature contractile apparatus, a hallmark of functional cardiomyocytes. This provides another qualitative metric supporting the validity of the proposed novel co-culture differentiation method. We repeated this assay using the AICS16 GFP_+ve_ cells and obtained similar qualitative results (Fig 4A, 4B and 4C).

To be certain that cardiomyocytes arising from AICS cells resulted from co-culture with iCMs, we performed an additional control experiment. Instead of co-culture exposure of AICS cells to IMR90-derived iCMs, AICS cells was co-cultured with IMR90 iPS cells. Similar to cells subjected to only media change, we could not observe any AICS11 or AICS16 cells co-cultured with IMR90 iPS cells to stain for sarcomeric α-actinin (S3 Fig). Consistently, we noticed that the fluorescence of GFP-β-actin was variably diminished in the majority of cells that stained positively for sarcomeres (S4 Fig), a phenomenon not observed for AICS11 cells expressing TOM20-GFP. The reduced GFP fluorescence in AICS16 cell-derived cardiomyocytes impacted our ability to track and hence to quantitate the efficiency of differentiation using GiWi or the co-culture protocols using microscopy-based methods. In the next section, we resorted to an alternate assay using in-situ staining for cardiomyocyte markers combined with flow cytometry to facilitate analysis of the entire co-culture population of cells.

### Co-cultured GFP-tagged cells express cardiac specific markers

**Fig 5:**
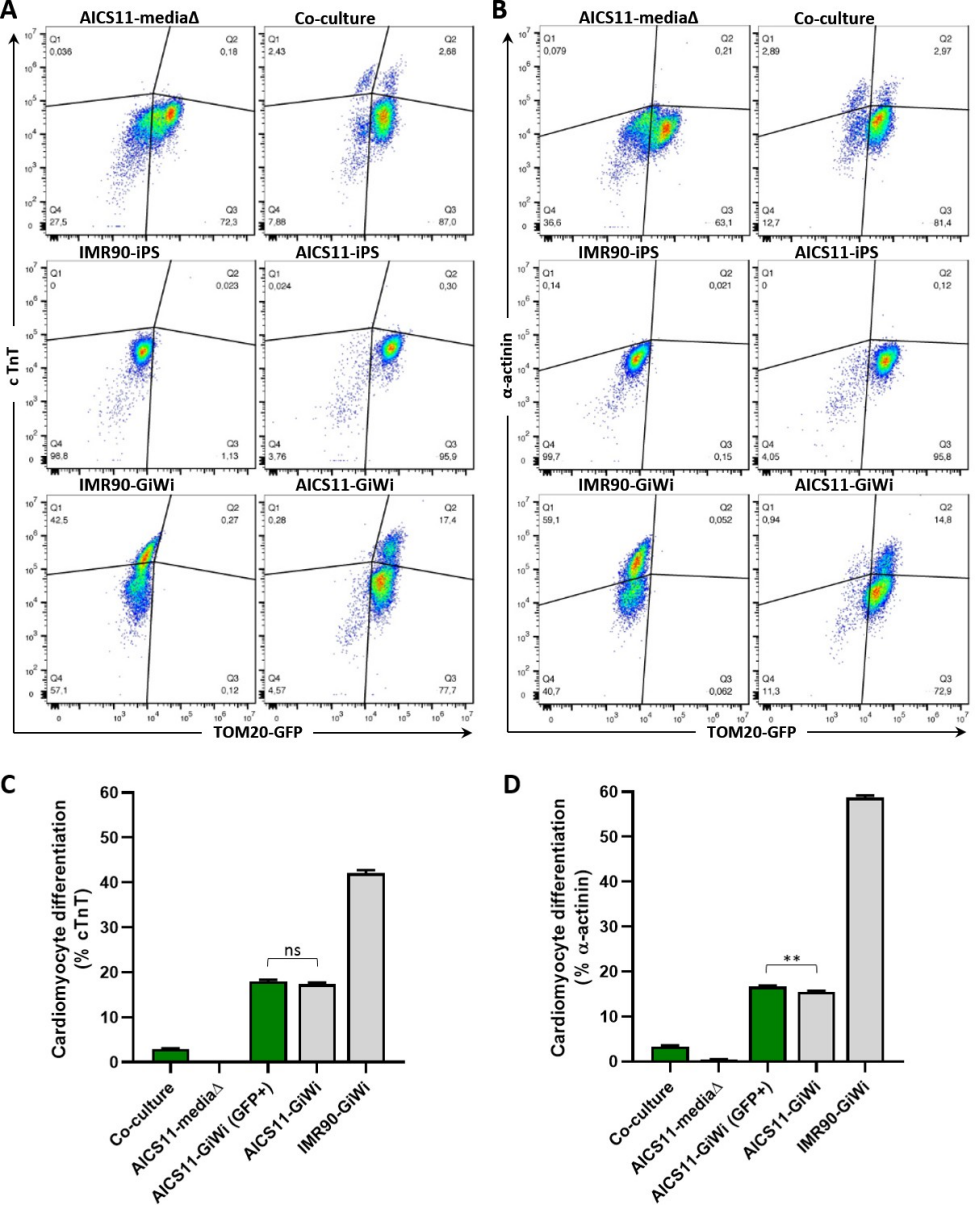
Cardiomyocyte differentiation efficiency of AICS11 co-cultured with iCMs evaluated by flow cytometry. AICS11 were either co-cultured with IMR90-iCMs, or subjected to media change only as a control. Additional controls include non-differentiated iPS or GiWi-differentiated AICS11 or IMR90 cells. Total cells were harvested and immuno-stained for the cardiomyocyte markers (A) cTnT and (B) a-actinin, and analyzed by flow cytometry in conjunction with TOM20-GFP to distinguish AICS11 cells. As shown are representative flow plots for an experiment performed in triplicates. Calculation of cardiomyocyte differentiation efficiency for (C) cTnT and (D) a-actinin. Green bars indicate cells gated for GFP only, where % = Q2/(Q2+Q3)*100, while grey bars indicate total cells, where % = (Q1+Q2)/(Q1+Q2+Q3+Q4)*100. P-value for all pairwise comparison is ≤0.0001, except otherwise indicated, where ns is non-significant, and ** is <0.002.

**Fig 6:**
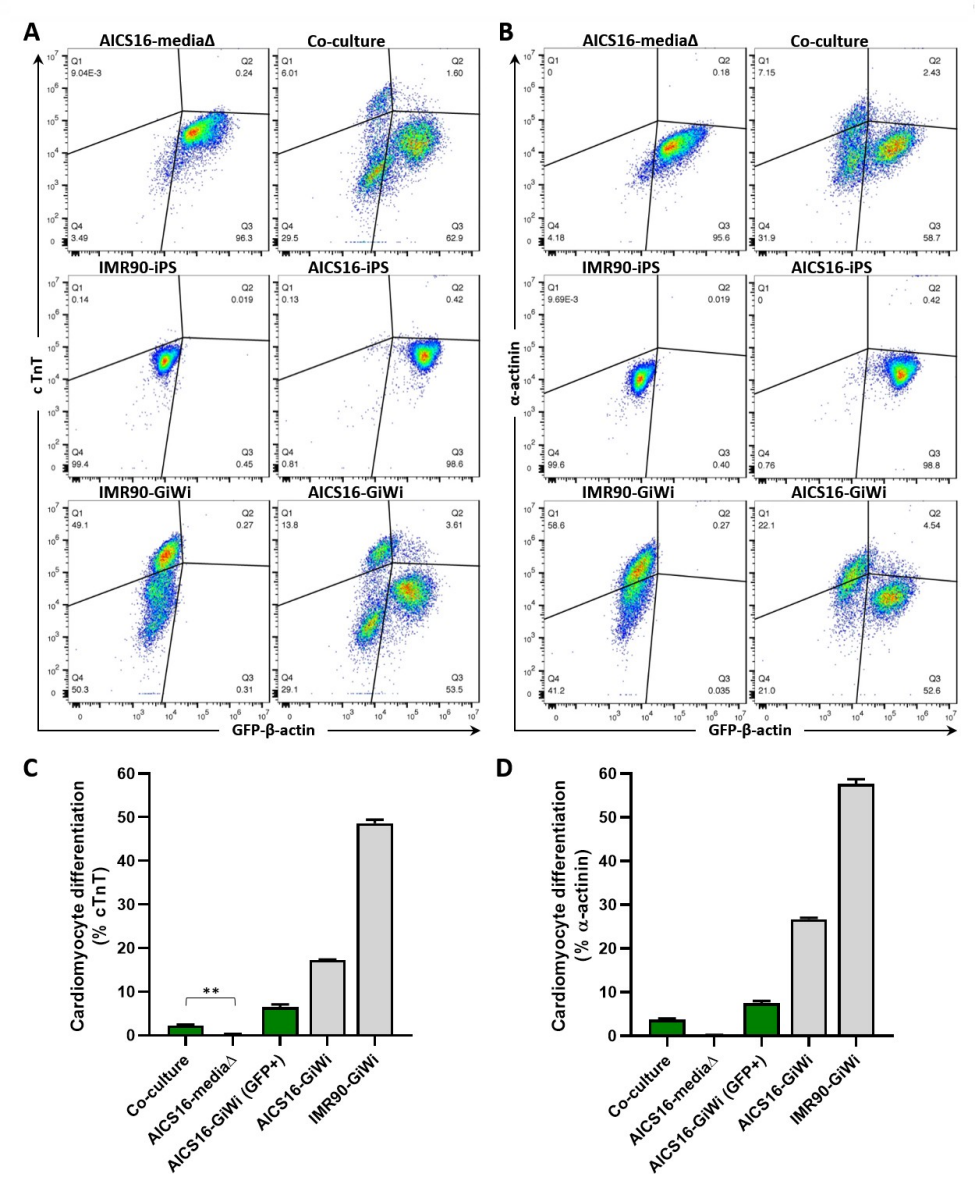
Cardiomyocyte differentiation efficiency of AICS16 co-cultured with iCMs evaluated by flow cytometry. Description for this experiment is identical to that for Fig 5, but with AICS16 cells (expressing GFP-b-actin) in place of AICS11. P-value for all pairwise comparison is ≤0.0001, except otherwise indicated, where ** is <0.003.

To obtain quantitative data assessing the efficacy of the co-culture method as a viable cardiac differentiation system, the differentiation efficiency for cells within the population was analyzed using flow cytometry. Differentiation efficiency was defined as the proportion of cells in a given population expressing either cardiac troponin T (cTnT) or α-actinin, while GFP fluorescence was used to delineate AICS11 (Fig 5A and 5B) or AICS16 (Fig 6A and 6B) cells from the non-fluorescent IMR90-derived iCMs. In addition to the co-culture experimental group, each flow experiment included the following controls to facilitate proper gating of the populations for final analysis; iPS and GiWi-differentiated IMR90 cells, iPS and GiWi-differentiated AICS11/16 cells, and AICS11/16 cells subjected to only media changes (not overlayed with iCMs).

Using the controls stated above, and gating for only GFP_+ve_ cells, flow cytometry analysis of co-cultured samples revealed that ~3.0% and ~3.3% of AICS11 cells were positive for cTnT and α-actinin, respectively (Fig 5C and 5D). We noted too that the differentiation efficiency by GiWi of AICS11 cells were at least 2-fold less than for IMR90 cells (cTnT:18% and α-actinin:17% for AICS11; cTnT:42% and α-actinin:59% for IMR90). However, GFP_+ve_ gating of GiWi-dfferentiated AICS11 cells accounted for the same efficiency when compared to the total population (16.7% GFP_+ve_ vs 15.5% total). In that regard, co-culture of AICS11 cells with IMR90 iCMs achieved approximately 18% (3/16.7*100) of the efficiency obtained by GiWi-differentiation of AICS11.

Using a similar analysis, AICS16 cells differentiated via co-culture had ~2.3% and ~3.7% of GFP_+ve_ cells that were positive for cTnT and α-actinin, respectively (Fig 6C and 6D). AICS16 cells differentiated via GiWi exhibited much lower GFP_+ve_ cells that were positive for CM markers (cTnT:6.5%; α-actinin:7.4%), compared to the total population (cTnT:17.2%; α-actinin:26.7%). As alluded to in the preceding section, AICS16 iPS cells differentiated into cardiomyocytes suffer from diminished GFP fluorescence, indicated by a leftward shift in the cTnT_+ve_ and α-actinin_+ve_ population of AICS16 GiWi-differentiated cells (Fig 6A and 6B). The diminished GFP signal in AICS16-differentiated cardiac cells translates to an eventual underestimation of differentiation efficiency of our co-culture method using AICS16 cells when gated purely for GFP_+ve_ cells. However, despite this limitation in gating that prevented a complete valuation of the actual differentiation efficiency of co-culture differentiation of the AICS16 cell line, flow data from AICS11 experiments (which does not suffer from a diminished GFP signal) provides statistically significant evidence showing detectable proportion of cells expressing cardiac specific markers when differentiated via our co-culture method.

## Discussion

The work presented here provide strong evidence that iPS cells can be differentiated into cardiomyocytes simply by inductive co-culture with an existing population of iPS-derived cardiomyocytes. Cardiomyocytes differentiated in this manner exhibit self-contractility and other distinguishing features of functional cardiomyocytes, including formation of sarcomeric striations and expression of cardiac specific markers, cTnT and α-actinin. Importantly, the successful reprogramming of iPS cells into CMs was achieved without the addition of exogenous pathway inhibitors or morphogens into the co-culture, suggesting that the ‘older’ seeded iCMs constituted a sufficient system that recapitulates the stimulatory environment for cardiac differentiation of iPS cells into relevant cardiomyocyte subtypes.

During optimization of the co-culture method, we had considered three mixing strategies. The first was the method described in the preceding sections, where iCMs were seeded onto already adherent iPS cells in culture. The second was a variation of the first but in reverse order, where iPS cells was seeded onto already adherent iCMs. The third strategy involved harvesting both iPS and iCMs, mixed together and seeded simultaneously onto fresh Matrigel coated plates. Of the three strategies, only the first method, where iPS cells were pre-cultured, yielded successfully differentiated cells, as evidenced using the criteria that cells of iPS origin can sustain spontaneous self-contractions when observed as isolated single cells.

It seems plausible that the ability for iPS cells to be differentiated into CMs require adequate cell adhesion to begin with. The second co-culture approach presented little adhesion surface for iPS cells that is also likely to be remodeled by the existing iCMs, while the third approach may have highlighted the competition between the co-seeded iPS and iCMs for limited adhesion surfaces. This ‘race to the surface’ phenomenon has also been documented in other co-culture experiments [15, 16], though not in the context of stem cells and stem cell differentiation. As per convention, the GiWi-protocol recommends initiation of differentiation when the iPS cell monolayer is at near confluence, suggesting the importance of preexisting iPS cell-cell and cell-ECM interaction. In this regard, the iCMs appear to tolerate physical manipulation, including the act of trypsin dissociation of the differentiated tissues followed by reseeding onto the iPS monolayer. The reseeded iCMs exhibit contractile behavior within several hours post reseeding on the iPS, providing a visual cue for successful integration onto a new ‘tissue’.

It should be noted that the quantity of iCMs introduced as an overlay were in excess of what could be integrated as an adherent tissue layer, with non-integrated cells removed during subsequent media replacement. This was predicated on initial optimization tests which indicated that the differentiation efficiency was proportional to the quantity of input iCMs.

It’s possible that greater efficiency of iPS to CM differentiation using inductive co-culture may be achieved. Our experimental setup relied on the ability to track the subject iPS cells being differentiated to CMs using a fluorescent label. To this end, we chose the CRISPR engineered AICS iPS cell lines from the Allen Institute for Cell Science, where GFP was stably integrated as fusion proteins. However, the bright fluorescence of GFP-β-actin of AICS16 became a liability, when it became clear that cells with AICS16-derived cardiomyocytes exhibit substantial loss of GFP-β-actin, preventing our ability to reliably assess differentiation efficiency in the co-culture. On the other hand, AICS11 iPS cells and cardiomyocytes expressed the mitochondrial TOM20-GFP protein at comparable levels. Side by side differentiation of IMR90 and both AICS iPS cell lines using GiWi suggested that the AICS derivatives achieved lower cardiomyocyte differentiation efficiency (50% or below that of IMR90). It is not clear if this also translated to a lower differentiation efficiency achieved via the inductive co-culture protocol. Ideally, both iCMs and iPS cells used in the co-culture should be in the same genetic background with high differentiation efficiency, in this case IMR90 cells with and without an integrated fluorescent protein label.

The successful iPS to CM differentiation using the pre-culture overlay method supports the postulate that direct cell-cell contact is essential in co-culture based cardiac differentiation. Previous works have also reported similar results [13, 17, 18], although not with iPS cells or with iCMs as a stimulatory source. Studies by Iijima *et al*. went a step further and demonstrated that not only is cell-to-cell contact essential to cardiac differentiation, but the cyclic contraction of neighboring cardiomyocytes is also necessary for cardiac reprogramming of adjacent cells [19]. It is pertinent to mention that our attempts at co-culture with iCMs seeded within a tissue culture insert, thus forming a physical barrier preventing direct iPS-iCM cell-cell contact but not the free exchange of soluble factors, also failed to induce any quantifiable CM differentiation (data not shown). With regards to molecular pathway, researchers are now trying to unravel the players involved in the intercellular coupling and communication governing this process. Of particular interest are gap junction proteins highly expressed in cardiac tissue such as connexin 43 (Cx43), which form low resistance conduction channels deemed essential for syncytia formation [20–22]. However, Cx43 may have additional roles facilitating cell-cell communication beyond its canonical role in electrical conduction. In this regard, opening of Cx43 hemichannels is implicated in extracellular release of ions and metabolites from the cytosol [23, 24]. Thus, it would be reasonable that gap junction like proteins such as Cx43 may facilitate direct exchange of programming factors between cells found in the iCM-derived tissue with iPS cells to induce differentiation toward a cardiomyocyte fate. Indeed, Cx43 was implicated in heterotypic cell interactions involving myofibroblasts and cardiomyocytes following a pathologic cardiac event [25], interactions that could influence functional cardiac tissue repair.

In summary, while highly efficient and convenient, the standard GiWi method for cardiomyocyte generation may fail for iPS cells derived from certain genetic backgrounds, in particular where GSK3 and Wnt pathway may be perturbed. In such circumstance, a benefit of using the proposed ‘near natural’ co-culture method for programming stem cell fate can serve to circumvent this challenge and generate physiologically relevant patient-specific cardiomyocytes.

## Supporting information

S1 Fig Fluorescent images and kymographs of co-culture differentiation of CellTracker™-labeled iPS cells via pre-culture method. (A) DIC image of pre-culture on day 8 (scale bar: 500μm), red box shows magnified overlay of DIC and TRITC images (scale bar: 100μm). Kymograph of contracting cluster indicated by red arrow line scan (scale bar: 50μm). (B,C) Overlay image of pre-culture at low density and kymographs showing fluorescently labeled cell exhibiting spontaneous self contraction (1) and non-contracting (2). Non-labeled cells can also be observed to exhibit spontaneous self contraction (3) and non-contracting (4). Scale bars: 50μm in overlay, 5μm in kymographs.
(PDF)

S2 Fig Fluorescent images and kymographs of co-culture differentiation of CellTracker™-labeled iPS cells via bi-culture method. (A) DIC image of pre-culture on day 8 (scale bar: 500μm), red box shows magnified overlay of DIC and TRITC images (scale bar: 100μm). Kymograph of contracting cluster indicated by red arrow line scan (scale bar: 50μm). (B) Overlay image of bi-culture at low density and (C) kymographs showing non-contractile fluorescently labeled cells (1, 2) and non-labeled cell exhibiting spontaneous self contraction (3). Scale bars: 50μm in overlays, 5μm in kymographs.
(PDF)

S3 Fig Staining of AICS16 (GFP-β-actin) and AICS11 (TOM20-GFP) cells for sarcomeric α-actinin. AICS16 or AICS11 cells were differentiated using (A,D) GiWi protocol, or (B,E) co-cultured with IMR90 iPS cells, and (C,F) basal media change alone (absent differentiation factors). Bottom panels show a magnified image of α-actinin staining for the area bounded by white rectangles.
(PDF)

S4 Fig GiWi-differentiated AICS16 cells exhibit diminished GFP-β-actin expression. (A) Overlay image showing α-actinin (red), GFP (green), and DAPI-stained cell nuclei (blue).(B) Fluorescent image showing only GFP (green). Cells staining for sarcomeric a-actinin (yellow arrows) exhibit reduced GFP fluorescence compared to neighbouring cells (green arrows).
(PDF)
