# Supplementary material for: Co-culture of induced pluripotent stem cells with cardiomyocytes is sufficient to promote their differentiation into cardiomyocytes
Source: PLoS One. 2020 Apr 3;15(4):e0230966. doi: 10.1371/journal.pone.0230966 (PMC7122760; doi:10.1371/journal.pone.0230966)
Supplement: S2 Fig — (A) DIC image of pre-culture on day 8 (scale bar: 500μm), red box shows magnified overlay of DIC and TRITC images (scale bar: 100μm). Kymograph of contracting cluster indicated by red arrow line scan (scale bar: 50μm). (B) Overlay image of bi-culture at low density and (C) kymographs showing non-contractile fluorescently labeled cells (1, 2) and non-labeled cell exhibiting spontaneous self contraction (3). Scale bars: 50μm in overlays, 5μm in kymographs. (PDF) [file pone.0230966.s002.pdf]

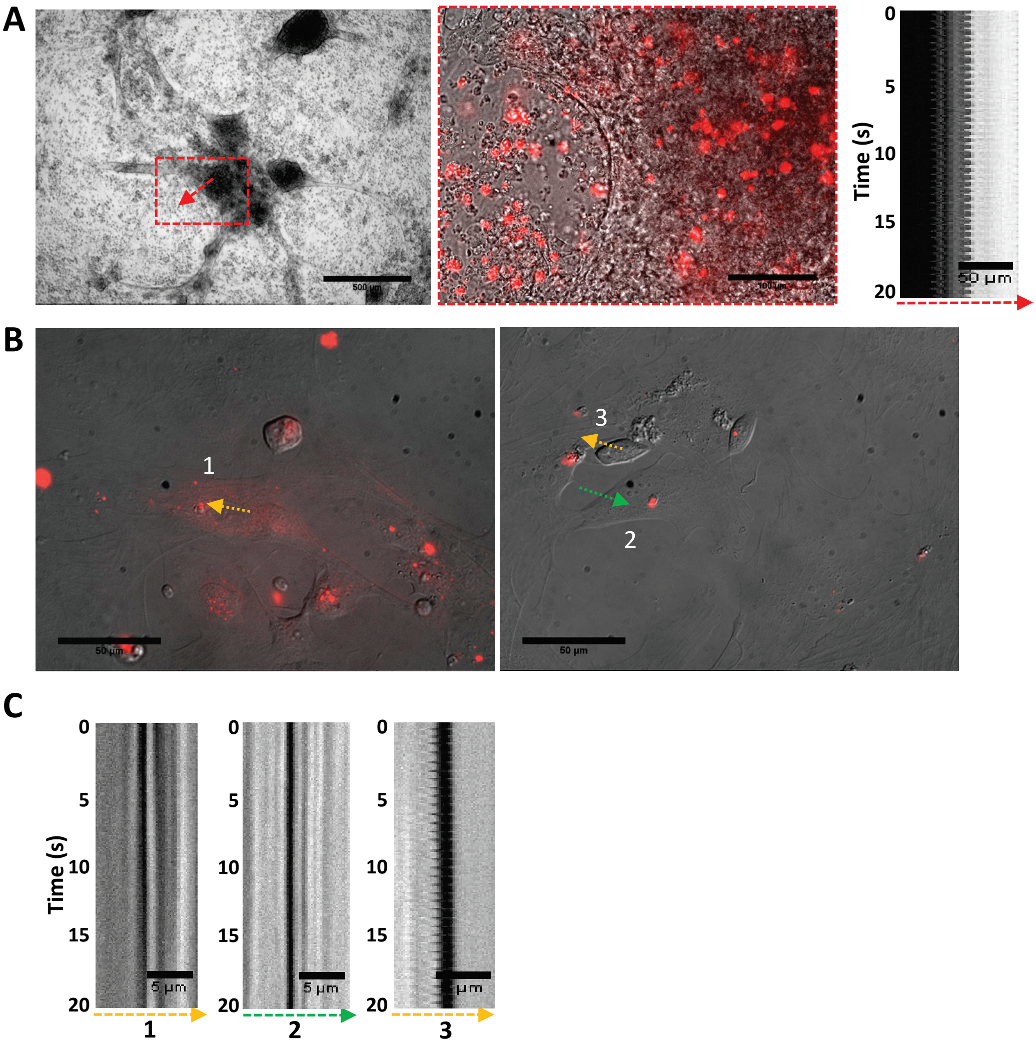

S2 Fig. Fluorescent images and kymographs of co-culture differentiation of CellTracker™-labeled iPS cells via bi-culture method.

(A) DIC image of pre-culture on day 8 (scale bar: 500µm), red box shows magnified overlay of DIC and TRITC images (scale bar: 100µm). Kymograph of contracting cluster indicated by red arrow line scan (scale bar: 50µm). (B) Overlay image of bi-culture at low density and (C) kymographs showing non-contractile fluorescently labeled cells (1, 2) and non-labeled cell exhibiting spontaneous self contraction (3). Scale bars: 50µm in overlays, 5µm in kymographs.
