# Supplementary material for: Co-culture of induced pluripotent stem cells with cardiomyocytes is sufficient to promote their differentiation into cardiomyocytes
Source: PLoS One. 2020 Apr 3;15(4):e0230966. doi: 10.1371/journal.pone.0230966 (PMC7122760; doi:10.1371/journal.pone.0230966)
Supplement: S3 Fig — AICS16 or AICS11 cells were differentiated using (A,D) GiWi protocol, or (B,E) co-cultured with IMR90 iPS cells, and (C,F) basal media change alone (absent differentiation factors). Bottom panels show a magnified image of α-actinin staining for the area bounded by white rectangles. (PDF) [file pone.0230966.s003.pdf]

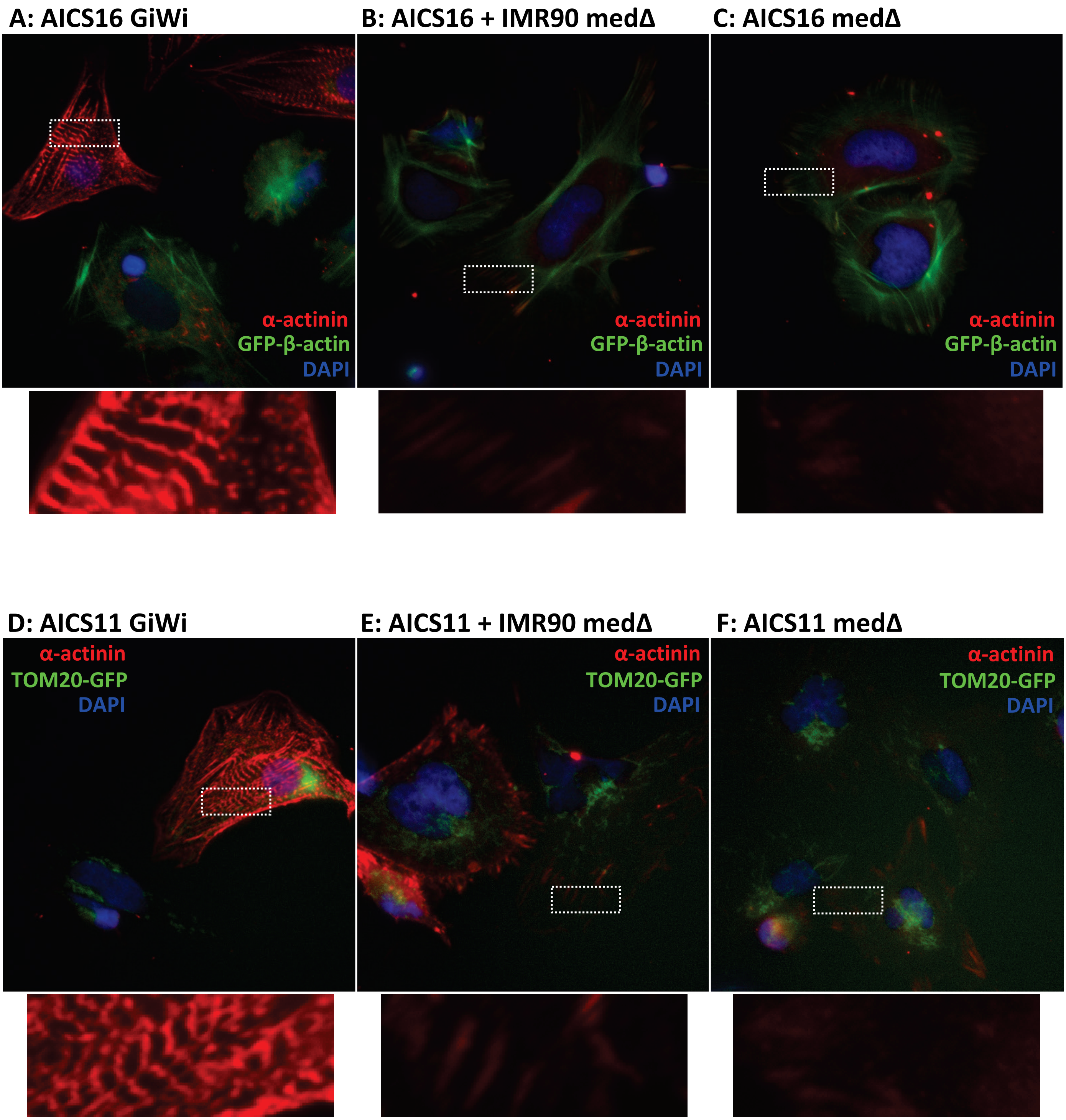

S3 Fig. Staining of AICS16 (GFP-β-actin) and AICS11 (TOM20-GFP) cells for sarcomeric α-actinin. AICS16 or AICS11 cells were differentiated using (A,D) GiWi protocol, or (B,E) co-cultured with IMR90 iPS cells, and (C,F) basal media change alone (absent differentiation factors). Bottom panels show a magnified image of α-actinin staining for the area bounded by white rectangles.
