# Supplementary material for: Co-culture of induced pluripotent stem cells with cardiomyocytes is sufficient to promote their differentiation into cardiomyocytes
Source: PLoS One. 2020 Apr 3;15(4):e0230966. doi: 10.1371/journal.pone.0230966 (PMC7122760; doi:10.1371/journal.pone.0230966)
Supplement: S4 Fig — (A) Overlay image showing α-actinin (red), GFP (green), and DAPI-stained cell nuclei (blue).(B) Fluorescent image showing only GFP (green). Cells staining for sarcomeric a-actinin (yellow arrows) exhibit reduced GFP fluorescence compared to neighbouring cells (green arrows). (PDF) [file pone.0230966.s004.pdf]

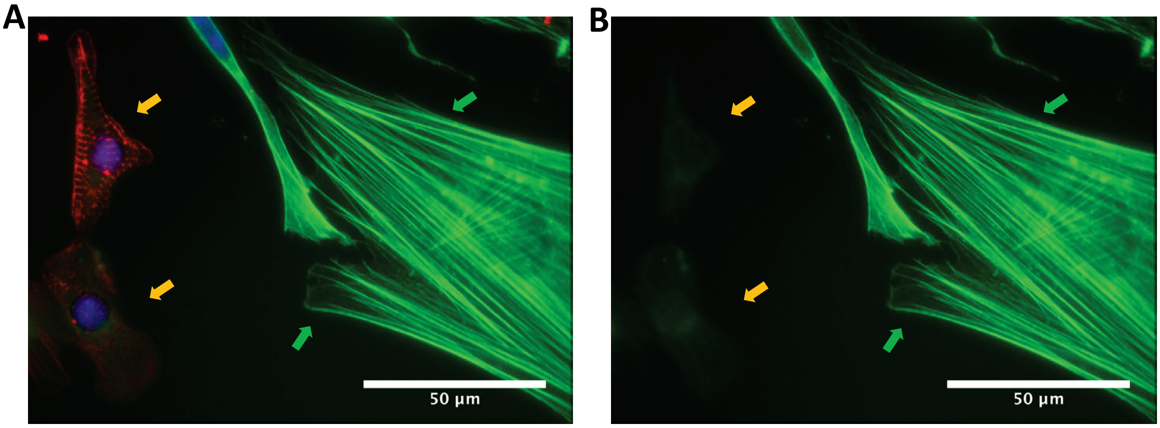

S4 Fig. GiWi-differentiated AICS16 cells exhibit diminished GFP-β-actin expression. (A) Overlay image showing α-actinin (red), GFP (green), and DAPI-stained cell nuclei (blue).(B) Fluorescent image showing only GFP (green). Cells staining for sarcomeric a-actinin (yellow arrows) exhibit reduced GFP fluorescence compared to neighbouring cells (green arrows).
